# Influence of Exogenous 24-Epicasterone on the Hormonal Status of Soybean Plants

**DOI:** 10.3390/plants12203586

**Published:** 2023-10-16

**Authors:** Michael Derevyanchuk, Serhii Kretynin, Yaroslava Bukhonska, Igor Pokotylo, Vladimir Khripach, Eric Ruelland, Roberta Filepova, Petre I. Dobrev, Jan Martinec, Volodymyr Kravets

**Affiliations:** 1 VP Kukhar Institute of Bioorganic Chemistry and Petrochemistry, National Academy of Sciences of Ukraine, 02094 Kyiv, Ukraine; 2 Génie Enzymatique et Cellulaire, UMR CNRS 7025, Université de Technologie de Compiègne, 60203 Compiègne, France; eric.ruelland@utc.fr; 3 Institute of Bioorganic Chemistry, National Academy of Sciences of Belarus, Kuprevich Str., 5/2, 220141 Minsk, Belarus; 4 Institute of Experimental Botany, The Czech Academy of Sciences, 16502 Prague, Czech Republic

**Keywords:** 24-epicastasterone, salicylic acid, auxins, hormones, soybean

## Abstract

Brassinosteroids (BRs) are key phytohormones involved in the regulation of major processes of cell metabolism that guide plant growth. In the past decades, new evidence has made it clear that BRs also play a key role in the orchestration of plant responses to many abiotic and biotic stresses. In the present work, we analyzed the impact of foliar treatment with 24-epicastasterone (ECS) on the endogenous content of major phytohormones (auxins, salicylic acid, jasmonic acid, and abscisic acid) and their intermediates in soybean leaves 7 days following the treatment. Changes in the endogenous content of phytohormones have been identified and quantified by LC/MS. The obtained results point to a clear role of ECS in the upregulation of auxin content (indole-3-acetic acid, IAA) and downregulation of salicylic, jasmonic, and abscisic acid levels. These data confirm that under optimal conditions, ECS in tested concentrations of 0.25 µM and 1 µM might promote growth in soybeans by inducing auxin contents. Benzoic acid (a precursor of salicylic acid (SA)), but not SA itself, has also been highly accumulated under ECS treatment, which indicates an activation of the adaptation strategies of cell metabolism to possible environmental challenges.

## 1. Introduction

Plant hormones are biologically active compounds produced by their cells, which have a relatively complex structure and affect certain chemical molecules in the process of their metabolism [1]. In recent years, research on the role of the representatives of a rather large class of steroid hormones, brassinosteroids, in the regulation of cell metabolism at the level of signaling systems has been developing at a rather rapid rate [2,3,4,5,6,7].

BRs, as a unique class of plant hormones, are involved in the regulation of plant growth and development [3,8], in particular, under the influence of stress factors [9,10]. Changes in the concentration of endogenous BRs [11,12,13] play an important role in the adaptation of plant metabolism. Exogenous BRs have been shown to increase growth [14], respiration rate [15], and plant resistance, in particular, under drought conditions [16] and pathogen attack [17]. BRs have a strong impact on photosynthesis and the membrane properties under acclimation to temperature stress [14]. BRs interact with the signaling pathways of other phytohormones [18], in particular those of abscisic acid (ABA) [19], cytokinins [20], auxins [21,22,23], gibberellins (GCs) [24,25], jasmonic acid (JA) [26], ethylene [27], salicylic acid (SA) [28] and other plant hormones [29,30,31].

The experimental data have also shown that exogenous BRs have a strong impact on endogenous BR biosynthesis. 24-epibrassinolide (EBL) at a low concentration 0.01–1 μM could increase the BR content in the leaves of barley plants [9]. Furthermore, EBL has effectively ameliorated endogenous BR level and plant growth suppressed by a specific inhibitor of BR biosynthetic reactions—brassinazole [9]. These studies point to complex interactions between different types of BRs and other phytohormones important for the fine-tuning of plant metabolism. To analyze the cross-interaction of brassinosteroids with other plant hormones, genetic approaches using transgenic plants and the exogenous effect of brassinosteroids on them have been widely applied [3,27,32]. Considerably less attention is paid to determining changes in hormone content in vivo in plants under the exogenous action of brassinosteroids [33]. The effects of BRs on plant cell metabolism have been described in numerous publications, but knowledge of its molecular mechanisms remains poorly studied. Further research opens up prospects for a more efficient use of BRs as an environmentally conscious regulator of plant growth and productivity [34].

The goal of the present study is to analyze the effect of exogenous 24-epicastasterone (ECS) treatment on the endogenous content of key phytohormones, in particular auxins (indole-3-acetic acid (IAA)), abscisic, salicylic, and jasmonic acid content in soybean leaves. ECS is a natural brassinosteroid phytohormone that attracts our attention mainly because it is a direct biosynthetic precursor of EBL [35], it is widely distributed in plants [36], and it plays an important role in the regulation of shoots and leaf growth [37]. Establishing the exogenous influence of ECS on changes in the content of other hormones is another level of analysis that can provide information that will help shed more light on the complex network of crosstalk between BRs and other hormones.

## 2. Results and Discussion

### 2.1. Interaction of Brassinosteroids with Auxins in Plant Cells

Auxins are strong inducers of plant cell metabolic reprogramming, causing the rapid acceleration of their growth and development, acting locally or through distinct signaling. Yet, auxins have some inhibiting role in roots growth. IAA is one of the major and most common auxins produced by plants, which stimulates plant growth [38,39].

**Figure 1:**
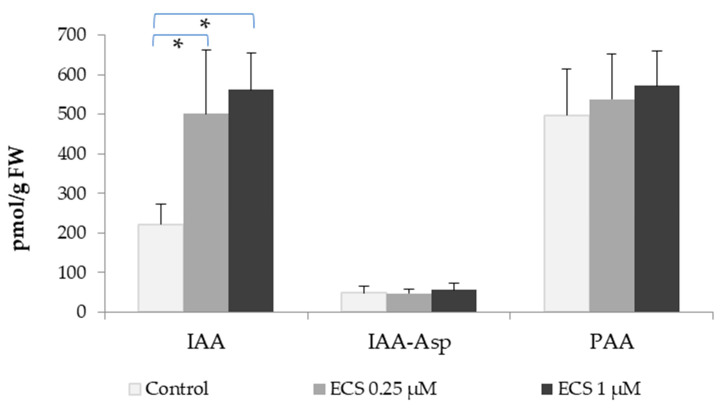
Influence of ECS treatment on endogenous content of auxins-indole-3-acetic acid (IAA), IAA ester with aspartate (IAA-Asp), and phenylacetic acid (PAA) in soybean leaf tissues. Plants have been sprayed twice with ECS on the 21st and 28th days after planting. On the 35th day after planting, soybean leaves were harvested to determine the phytohormone content. Error bars indicate standard error of the mean (SE). * *p* < 0.05 (Student’s *t*-test).

In our research, we detected the levels of IAA, phenylacetic acid (PAA), and IAA ester with aspartate (IAA-Asp) in soybean leaf tissues. The levels of auxins under control conditions were measured to be around 221.9 pmol/g FW for IAA, 48.5 pmol/g FW for IAA-Asp, and 497.1 pmol/g FW for PAA. The treatment of soybean plants with ECS solution (0.25 µM, or 1 µM) caused a strong increase in IAA content (Figure 1). In contrast, no significant changes in IAA-Asp and PAA content have been observed in soybean leaf tissues treated with the indicated concentrations of ECS (Figure 1). PAA is a natural auxin [40] found in a number of plants [41], but to date, information on its distribution metabolism and function in plants is still limited, and our data on the response of PAA content to the effects of ECS on plants is a contribution to the study of its role in plants (Figure 1). According to our results, the content of PAA in soybean leaves exceeded more than twice the content of IAA (Figure 1); a higher content of PAA compared to IAA was also found in a number of other plants, and among the possible explanations is higher activity and mobility in the tissues of IAA compared to PAA [41]. A significant increase in the content of IAA was earlier shown in rice treated with EBL under salinity conditions. These changes correlated with enhanced plant growth under salt stress action [42]. In contrast, no induction of IAA content by EBL was reported under optimal growth conditions [43]. In another report, brassinolide (BL)—another type of BR—promoted auxin content in *Arabidopsis* roots but inhibited auxin signaling [43]. Moreover, the inhibition of BR signaling in the outer tissues of roots results in a meristem insensitivity to both BRs and auxin [43]. In our study, exogenous ECS under optimal conditions stimulated IAA content (Figure 1). This might point to the importance of the particular BR species and concentrations used in evoking dissimilar responses in different plant species. However, we observed no significant changes in IAA-Asp or PAA content; our data are the first observation of IAA-Asp and PAA levels in response to BR.

One of the key interactions between BRs and auxins can be seen in their mutual effect on the regulation of gene expression. BRs can regulate the expression of the *AtYUC9* gene involved in auxin biosynthesis [43], while auxins can modulate the expression of genes involved in BR biosynthesis [44,45]. BRs and auxins also interact at the cellular level. BRs can promote the transport of auxins by modulating the expression of auxin transporters *PINFORMED3 (PIN3)* and *PIN4* in *Arabidopsis* [46], and auxin responsive genes such as *IAA18*, *IAA30*, and Auxin Response Factor12 in wheat [47], while auxins can affect BR signaling by regulating the expression of BR receptors [48].

Via the Genevestigator array data analysis, we could see that the expression level of several genes involved in auxin biosynthesis and signaling is modified in response to ECS or BL treatment in soybean (*Glycine max*) [49] (Table 1). The expression of soybean genes encoding FLAVIN-CONTAINING MONOOXYGENASE increased under ECS treatment (Table 1). Treatment with ECS (12 h), propiconazole (an inhibitor of BR biosynthesis), or combined treatment overall decreased the expression of genes coding auxin transporters or auxin signaling components in the roots of soybean (Table 1). In contrast, BL treatment in leaves strongly induced the same genes (Table 1). This indicates that the hormone concentration, its type, and the site of treatment have a strong impact on the outcome of gene expression.

Auxins are involved in the metabolism reprogramming of plant cells, causing the acceleration of their growth and development [50,51]. The overexpression of PIN-LIKES auxin transporters in *imp* mutants strongly influences BR signaling, possibly by changing local auxin levels [52]. Auxins and BRs stimulate growth and affect the elongation of the hypocotyl, but the hormone crosstalk mechanism implicated in this process is not fully clear [39,53]. BRs also stimulate auxin levels important for the induction of lateral roots grown under low nitrogen stress by inducing the *AtYUC5*, *AtYUC7*, and *AtYUC8* auxin biosynthetic genes. In the BR signaling mutants *bsk3,4,7,8,* and *bzr1*, the expression of the above-mentioned *YUC* genes was not upregulated by low nitrogen conditions anymore. Interestingly, in *bzr1-1D* mutant plants, which have a stabilized variant of the BR-dependent transcription factor BRASSINAZOLE RESISTANT 1 (BZR1), the auxin genes *AtYUC7* and *AtYUC8* were upregulated constantly, independently of low nitrogen conditions [22]. This clearly points to a close relation between BR and auxin biosynthesis at the genes level. Interactions between BRs and auxins play a pivotal role in the regulation of several other key aspects of plant growth and development [52,54,55,56].

Our results on the ECS-dependent upregulation of IAA levels in soybean leaves are consistent with many published research articles about BRs role in the regulation of IAA signaling [32] in plant cells. It has been shown that an exogenous BR under low temperatures induces the content of endogenous IAA in cucumber seedlings, while the inhibitor of BR synthesis, brassinazole, decreased levels of IAA [33].

### 2.2. Interaction of Brassinosteroids with Plant Cell Stress Hormones

#### 2.2.1. Interaction of Brassinosteroids with Abscisic Acid

The results of microarray studies indicate that BRs and ABA provide a general regulation of the expression of hundreds of different genes involved in metabolic regulation; however, not many of them have overlapped [57,58]. At the same time, the key molecular mechanisms and components of signaling systems involved in these phytohormone interactions require further studies.

**Figure 2:**
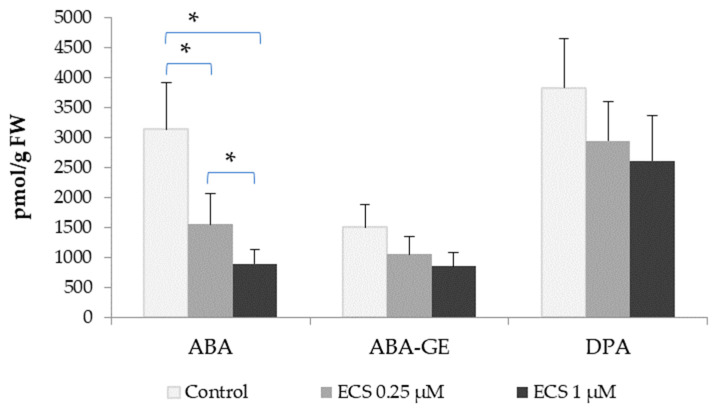
The effect of ECS on abscisic acid (ABA) and its metabolite levels: ABA-glucose ester (ABA-GE) and dihydrophaseic acid (DPA). Plants were sprayed twice with ECS on the 21st and 28th days after planting. On the 35th day after planting, soybean leaves were harvested to determine their phytohormone content. Error bars indicate standard error of the mean (SE). * *p* < 0.05 (Student’s *t*-test).

In the present study, we analyzed the contents of abscisic acid and its inactive metabolite, dihydrophaseic acid (DPA), in soybean leaves. The ABA level of the control plants was 3138.5 pmol/g FW, its glucose ester (ABA-GE)—1501 pmol/g FW, and DPA—3826.5 pmol/g FW (Figure 2). An exogenous treatment with ECS decreased the ABA and ABA-GE levels in soybean leaves (Figure 2). We did not observe any significant changes in the DPA levels of the treated plants (Figure 2). BRs have previously been shown to reduce ABA accumulation, resulting in the inhibition of stomatal closure induced by ABA [59]. The foliar application of EBL significantly reduced the level of abscisic acid in rice plants under stress or optimal conditions [42]. Moreover, freezing conditions in tolerant barley lines themselves promote the endogenous level of homocastaterone (BR type) and decrease the ABA level [13]. BRs and ABA antagonistically regulate many key developmental processes such as germination and seed maturation [60,61]. The BR biosynthetic mutant, *det2-1*, and the BR signaling mutant, *bri1-1*, are more sensitive to the ABA inhibition of seed germination than the wild-type. These earlier observations also pointed out that the germination rate in the mentioned mutants was not affected [62]. However, further investigations with BR mutants *det2-1* and *bri1-301* have found a strong reduction in seed germination in mutant and wild-type lines by exogenous ABA [58]. The overexpression of the BR receptor BRI1 strongly increased the germination rate of seeds treated with ABA [53]. The involvement of BRI1-associated receptor kinase 1 (BAK1) in the regulation of ABA signaling during seed germination and primary root growth has been shown in *Arabidopsis* [63]. Moreover, the inhibited germination of wild-type and *det2-1* lines, but not that of *bri1-301*, by ABA can be rescued by BL treatment [58]. It is known that the effects of ABA in seeds are associated mainly with the prevention of premature germination [64]. In contrast, BRs attenuate the effect of ABA and promote seed germination as well as vegetative growth and the development of plants [19,65]. BRs antagonize ABA-mediated responses in plants through a family of transcription factors, BZR1, which can reduce the expression of the main ABA-signaling component, ABA INSENSITIVE 5 (ABI5) [65]. Furthermore, it has been found that BZR1 directly binds to the E-box sequences in the *AtABA2* promoter region, decreasing the level of endogenous ABA in *A. thaliana* [19]. Treatment with brassinazole (Brz), a biosynthetic inhibitor of BR synthesis, increased *AtABA2* expression, an effect that can be attenuated by the exogenous use of BL [19]. The results of the studies performed by J. Moon and colleagues and our results on the use of ECS in soybeans (Figure 2) indicate a key role of BRs in the regulation of ABA levels in plants [19].

At the whole-plant level, BRs and ABA can act also synergistically depending on the specific physiological process. For example, under salt stress, EBL positively regulates the expression of ABA biosynthesis genes (*OsNCED1*, *OsNCED2*, *OsNCED3*, *OsNCED4*, *OsNCED5*, and *OsZEP1*) and two catabolic genes, *OsBG2* and *OsABAox3*. in rice [36]. Owing to these specific cell processes, ABA regulates the response to environmental stresses (for complete review see [66]). ABA, through the transcriptional factor ABSCISIC ACID INSENSITIVE3 (ABI3), slightly induces the BR biosynthesis regulatory gene *OsGSR1*, which evidences ABA’s role in the stimulation of BR production [67]. Moreover, ABA promotes the expression of key BR biosynthesis genes *AtDWF4* and *AtCPD*, but for effective ABA–BR crosstalk, the BIN2 signaling component is required [58]. Interestingly, while ABI3 promotes BR biosynthesis in *Arabidopsis*, the BR-regulated transcription factor BES1 can directly bind another ABA transcription factor, ABSCISIC ACID INSENSITIVE5 (ABI5), which reduces ABI5-regulated genes and suppresses the ABA response [68]. This suggests that BR-ABA antagonism and synergism on the level of hormone biosynthesis and signaling can be achieved by different transcription factors but the conditions that impact these processes require further elucidation. Seed germination is an essential stage of plant development that is regulated by various endogenous signaling systems in terms of its interaction with environmental factors [69].

#### 2.2.2. Effect of 24-Epicastasterone on Salicylic and Benzoic Acids Levels

**Figure 3:**
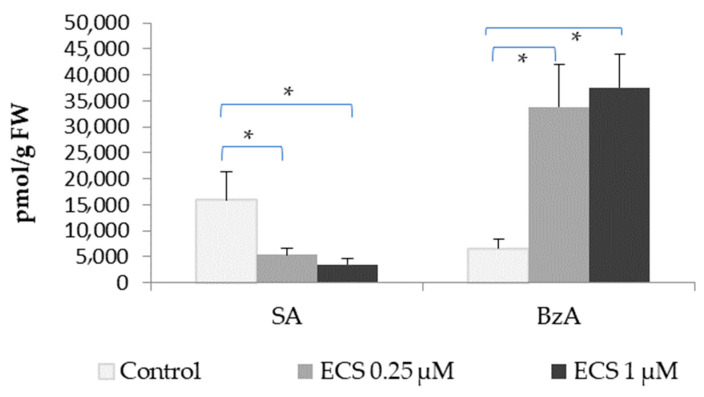
The effect of ECS on the level of salicylic (SA) and benzoic (BzA) acid in soybean leaf tissues. Plants have been sprayed twice with ECS on the 21st and 28th days after planting. On the 35th day after planting, soybean leaves were harvested to determine the phytohormone content. Error bars indicate standard error of the mean (SE). * *p* < 0.05 (Student’s *t*-test).

In the soybean plants exposed to ECS, the content of SA decreased while the content of BzA increased significantly, and in both cases, there was not a significant difference between the studied ECS concentrations (Figure 3). Recent studies on hormone levels under optimal conditions observed that freezing-tolerant barley lines accumulate significant levels of SA but not BRs (homocastasterone was analyzed in the paper). In contrast, under cold stress conditions, tolerant lines accumulate more homocastasterone, while the SA content significantly drops [13]. These data are consistent with our results (Figure 3) and might point to BRs’ and SA’s role in adaptation processes to abiotic stresses.

The BR and SA signaling networks are known to be, at least in part, interconnected. BRs can act synergistically and promote SA signaling by inactivating BR-INSENSITIVE 2 (BIN2) in *Arabidopsis*. BIN2 was shown to phosphorylate the TGA4 transcriptional factor (TF), which leads to its destabilization and prevents its functional interaction with NRP1 in mediating SA-regulated gene expression [70]. Furthermore, EBL has been shown recently to induce SALICYLIC ACID-BINDING PROTEIN2 in wheat [47]. Moreover, because clade I TGAs (a group comprising TGA4) have a role in regulating SA biosynthesis by promoting the expression of SARD1 and CBP60g [71], BRs, via the reported BIN2 phosphorylation of TGA4, can thus act in SA biosynthesis control. Moreover, in *Arabidopsis* lines overexpressing BAK1, an increase in endogenous SA accumulation was reported, suggesting that at least some components of the BR signaling cascade have a role in controlling SA accumulation [72].

BRs can also act antagonistically with SA. Based on the published data, the effect of BRs in controlling SA accumulation differs between plant species. Rice plants treated with BL could accumulate less SA during brown planthopper infestation; this effect was attributed to the downregulation of SA biosynthesis genes—*OsPAL* and *OsICS1* [71]. However, the latest data indicate that the phenylalanine ammonia-lyase (PAL) pathway does not directly lead to SA biosynthesis but has an important role in its regulation. [73]. Furthermore, BL was shown to reduce the SA content in tobacco [74]. The *Bsk1* plants, which are deficient in BR-SIGNALING KINASE1 (BSK1), accumulated less SA following an infection with *G. cichoracearum* powdery mildew or *Pseudomonas syringae* pv *tomato DC3000*). In addition, the *bsk1* mutant’s resistance to pathogens was compromised [75]. These data suggest that pathogen-induced SA accumulation relies on some components of the BR signaling cascade. SA can be produced via the isochorismate synthase (ICS) pathway (for review see [76]). In different plant species, the two pathways contribute unequally to SA biosynthesis. In *Arabidopsis*, most SA is produced via the ICS pathway independently of BzA accumulation [77]. In soybeans, both pathways, ICS and PAL, contribute equally to stress-induced SA accumulation [78]. The fact that an increase in BzA following ECS treatment is not translated into SA accumulation may imply that BA2H is inactivated. More so, this is because the pool of active SA in plants depends not only on the activity of SA biosynthetic pathways but also on the rate of SA conversion to inactive metabolites (for review, see [76]). Recently, important results for understanding the pathways of SA synthesis in plants were obtained by Wu and colleagues [73], who used stable isotopes (^13^C_6_-Phe and ^13^C_6_-BzA) to investigate the pathways of SA biosynthesis in *Arabidopsis*. They provided evidence that SA can be formed from benzoic acid, yet independently of the phenylalanine ammonia-lyase (PAL) pathway [73].

Our data on the accumulation of BzA (Figure 3) may evidence an important step in BR–SA crosstalk via the regulation of SA/BzA levels. Our results suggest that ECS induces an increase in BzA levels in soybeans, possibly via the inactivation of BA2H. Later, a BzA pool may enable a rapid conversion to SA and the activation of SA signaling. How SA biosynthesis is regulated by BRs remains to be established.

#### 2.2.3. Interaction of Brassinosteroids with Jasmonates (Jasmonic Acids and Jasmonic Acid-Isoleucine Levels)

**Figure 4:**
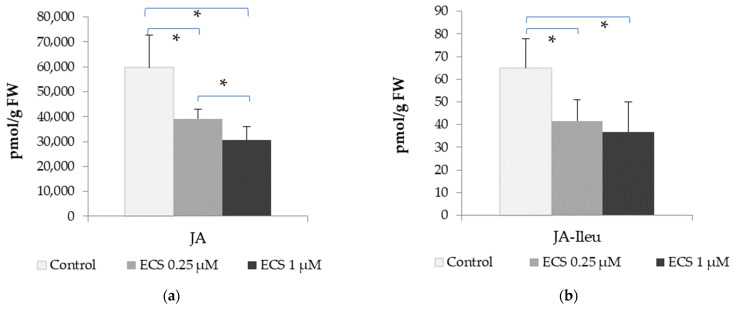
The effect of ECS on the level of (**a**) jasmonic acid (JA) and (**b**) the conjugate with isoleucine amino acid (JA-Ile) in soybean leaf tissues. Plants were sprayed twice with ECS on the 21st and 28th days after planting. On the 35th day after planting, soybean leaves were harvested to determine the phytohormone content. Error bars indicate standard error of the mean (SE). * *p* < 0.05 (Student’s *t*-test).

In the present study, we analyzed the content of JA and a JA conjugate with isoleucine (JA-Ile). The amino acid conjugate of isoleucine with JA is one of the common forms of JA in plant cells [79]. The content of JA in the control variant was determined in soybean leaves to be at the level of 65.03 pmol/g of FW and the JA-Ile content—59,636 pmol/g FW. Under the exogenous treatment of plants with 0.25 µM of ECS, a decrease in the JA and JA-Ile content was recorded. Higher concentrations of ECS (1 µM) did not lead to a further decrease in the levels of JA and JA-Ile (Figure 4).

An analysis of array data on the genes involved in jasmonate biosynthesis also revealed genes with modified expression in response to ECS or BL treatment [49] (Table 1). Genes that encode JASMONIC ACID-AMIDO SYNTHETASE JAR1 are highly suppressed in soybeans via treatment with exogenous ECS and BL (Table 1). In contrast, propiconazole (a BR-biosynthesis inhibitor) decreased their expressions (Table 1). JAR1 catalyzes the formation of JA conjugates with amino acids. These results are consistent with our data on ECS’s influence on the JA and JA-Ile levels (Figure 4). It has been shown previously that BR biosynthesis or BR signaling mutants show a higher accumulation of JA-precursor 12-oxo-phytodienoic acid [80], pointing to a BR–JA antagonism in the regulation of JA content. Furthermore, JA inhibits root growth and the expression of the BR biosynthetic gene *AtDWF4* and lowers the endogenous BR content, while exogenously added BRs can attenuate JA’s inhibitory effects on root growth [81]. These data are also consistent with the results that we obtained (Figure 4).

Some studies point to a concentration-dependent interconnection between BR and JA antagonism/synergism switches in different parts of the plant. For example, high levels of exogenously applied EBL in rice shoots have been demonstrated to induce the expression of the JA biosynthetic gene *OsAOS2* and the signaling gene *OsJAmyb* in plant roots. This has also been connected with the decreased activity of the BR biosynthetic *OsDwarf* and signaling *OsBRI1* genes. In contrast, low exogenous levels of EBL inhibited the *OsAOS2*/*OsJAmyb* genes. These changes in the JA and BR gene activities correlated with increased nematode resistance in plant roots, while the low exogenous concentration of EBL decreased plant resistance to nematode attack [80]. A major portion of BR–JA antagonism depends on the common component of the BR and JA pathways—BIN2 kinase. BIN2 kinase negatively regulates BR signaling by inactivating the BR-dependent transcriptional factors BRI1-EMS-SUPPRESSOR1 (BES1) and BZR1. On the other hand, BIN2 positively regulates JA signaling by phosphorylating and promoting the degradation of JAZ proteins and repressors of JAZ signaling. BR treatment represses BIN2 activity and the JA signal pathway. Gain-of-function mutations of BIN2 promote JA signaling and also stimulate JA accumulation [82]. The rice BIN2 homolog OsGSK2 has been shown to interact directly with a JA repressor protein, OsJAZ4, and positively regulate JA signaling and antiviral defense against rice black-streaked dwarf virus (RBSDV). BIN2 kinase is also a part of BR–JA synergism. OsGSK2 kinase can not only stimulate JA-signaling but also act as a negative regulator through interaction with the JA transcription factor OsMYC2. OsMYC2 phosphorylation by OsGSK2 leads to OsMYC2 degradation and a reduction in the JA-mediated defense response against rice stripe virus (RSV). It has been shown that RSV suppresses BR endogenous levels to elevate the accumulation of OsGSK2 [83]. So, at least in the case of plant responses to RSV, BRs and JA can act synergistically in the inhibition of the OsGSK2 kinase by BR. This inhibition promotes OsMYC2 activity and JA-dependent defense responses.

All the discussions above evidence an important role of BR–JA crosstalk in the regulation of plant cell metabolism. A cross-interaction between the JA and BR signaling pathways might be involved in the tight balancing between plant growth and resistance. The data we obtained suggest a BR–JA antagonism in soybean plant leaves under optimal conditions in response to treatment with exogenous ECS (Figure 4).

### 2.3. Effect of 24-Epicastasterone on Soybean Seed Weight

**Figure 5:**
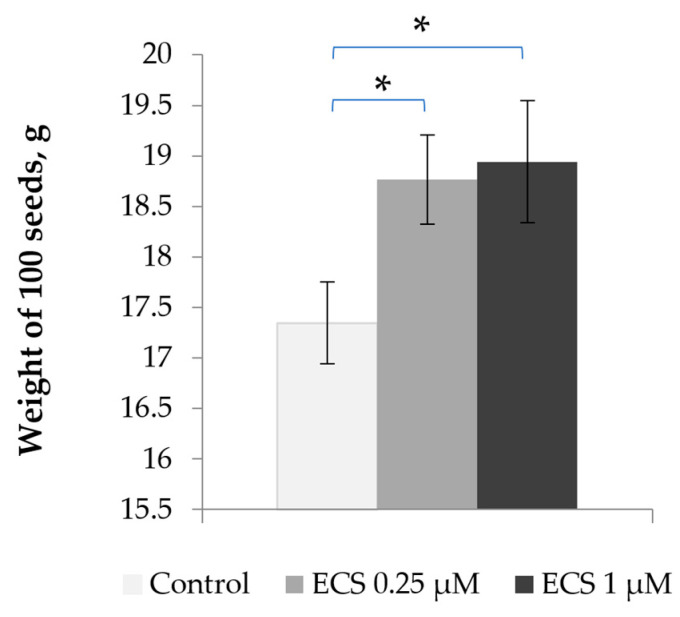
Influence of ECS treatment on average weight of soybean seeds. Plants were sprayed twice with ECS on the 21st and 28th days after planting. A batch of 100 seeds was measured. Error bars indicate standard error of the mean (SE). * *p* < 0.05 (Student’s *t*-test).

In our study, we evaluated the effect of exogenous ECS on soybean seed productivity as a long-term effect of ECS cross-hormonal interactions. We measured that the average weight of 100 seeds from the control plants corresponded to around 17.5 g, while the treatment of soybean plants with an ECS content of 0.25 or 1 µM led to an increase in the seed weight to almost 19 g for 100 seeds, an increase of about 8.5% (Figure 5). Thus, the observed changes in the content of key soybean hormones induced by ECS treatment are linked with an increase in soybean seed productivity.

## 3. Materials and Methods

### 3.1. Plant Materials and Growth Conditions

Soybean seeds (*Glycine max* L. cv. Terek) were obtained from the experimental station of Poltava State Agricultural University (Ukraine). Seeds were sterilized by being soaked in a 2.8% sodium hypochlorite (NaClO) solution for 5 min and then rinsed three times with autoclaved distilled water for 2 min each, germinated on sterile filter paper, and placed in 3 L plastic vessels filled with a growth substrate mixture—Polissya soil (70%), washed sand (0.5–2.0 mm) (15%), and agroperlite (15%).

Plants were grown at a constant temperature of 25 °C in an artificial climate chamber (14 h light: 10 h dark) under white light, which was provided by a continuous wide-spectrum LED (Philips) at a 250 μmol m^−2^ s ^−1^ intensity. The leaves of soybean plants grown in a vegetation experiment were sprayed twice with ECS solutions (0.25 or 1 µM) on the 21st and 28th days after planting the seedlings. On the 35th day after sowing, the soybean leaves were sampled from one tier of ten different plant vessels, mixed within each variant, quickly crushed, and four samples of 100 mg of leaf tissue were selected and frozen in liquid nitrogen for further determination of phytohormone content.

### 3.2. Treatment of Soybeans with ECS Solutions

ECS powder was synthetized in the laboratory of Volodymyr Khripach, an expert in the synthesis of different BR types and many of their derivatives. The ECS was dissolved in EtOH to obtain a 0.5 mM stock solution. The stock was diluted with distilled water to obtain the 0.25 and 1 μM final solutions used for soybean treatments. The ECS concentrations of 0.1 and 0.25 μM were chosen based on preliminary experiments with BR effects on soybeans and the available published data on BR usage on different species. The leaves of soybean plants grown in a vegetation experiment in plastic vessels were sprayed twice with ECS solutions (0.25 or 1 µM) on the 21st and 28th days after planting the seedlings using a hand sprayer; control plants were sprayed with a solvent, EtOH, diluted with water as ECS stock. The EtOH concentration in the final ECS solutions or control treatments did not exceed 0.01%.

### 3.3. Hormone Extraction and Quantification

Frozen samples (100 mg FW) were homogenized with liquid nitrogen in a mortar and pestle. The phytohormones were extracted with a cold (−20 °C) methanol/water/formic acid mixture (15/4/1, *v*/*v*), as described in [84]. Internal isotope-labeled standards (10 pmol per sample) were added for hormone analysis: ^13^C_6_-IAA (Cambridge Isotope Laboratories, Tewksbury, MA, USA); ^2^H_4_-SA (Sigma-Aldrich, St. Louis, MO, USA); ^2^H_3_-PA, ^2^H_3_-DPA, ^2^H_5_-ABA-GE (NRC-PBI, Saskatoon, SK, Canada); and ^2^H_6_-ABA, ^2^H_5_-JA, and others (Olchemim, Olomouc, Czech Republic). The extracts were passed through reversed-phase cation exchange SPE columns (Oasis-MCX, Waters, Milford, MA, USA) in a mixed mode (mixed phase–cation exchange). The hormone fraction containing ABA, IAA, SA, and JA was eluted with methanol. Hormone metabolites were analyzed using HPLC (Ultimate 3000, Dionex, Sunnyvale, CA, USA) coupled to a hybrid triple quadrupole/linear ion trap mass spectrometer (3200 Q TRAP, Applied Biosystems, Waltham, MA, USA). The quantification of hormones was carried out using the isotope dilution method with multilevel calibration curves (R^2^ > 0.99). Data processing was carried out with Analyst 1.5 software (Applied Biosystems).

### 3.4. Estimation of Average Weight of Soybean Seeds

To evaluate the effect of the ECS treatment on the average weight of soybean seeds, plants were harvested by hand and pods were separated from each plant. The pods were dried in an oven at 35 °C for 24 h to maintain homogeneous seed moisture, as described by Poudel [85]. A batch of 100 seeds was measured in one sample. Five biological samples per variant were analyzed.

### 3.5. Statistical Analysis

*p* values were calculated with a two-tailed Student’s *t*-test using Excel 2016 software. The sample size and number of independent biological repeats for each type of analysis are provided above.

## 4. Conclusions

The study of the ways of hormonal signaling in the regulation of plant metabolism is being carried out today with the use of a wide range of modern methodological approaches. The analysis of the effect of exogenous brassinosteroids on the content of key hormones in plant tissues is aimed at further improving the knowledge of the mechanisms of hormonal cross-interactions in the regulation of the metabolism of biological compounds in plant cells. A more complete picture of the molecular mechanisms underlying such interactions of phytohormones is an important task necessary to evaluate the complex and extensive network of hormonal signals in plants, as well as its spatial and temporal features. This process is complex and dynamic and requires further research at different levels of the organization of living systems, both in model plants and in important crops.

## Figures and Tables

**Table 1:** Analysis of Genevestigator array data of BR inhibitor propiconazole, ECS, and BL impact on the expression of genes involved in auxin and jasmonate biosynthesis and signaling in soybeans (*Glycine max*). Values are shown as the fold change of gene expression.

	Propiconazole/Untreated Seedlings **	Propiconazole/Untreated Roots ***	ECS/Untreated Roots ****	Propiconazole + ECS/Propiconazole *****	Propiconazole + BL/Propiconazole ******	Propiconazole + BL/Propiconazole *******
Auxin biosynthesis genes						
Flavin-containing monooxygenase (*GLYMA_10G128700*) *	1.02	2	1.42	−1.95	−1.02	1.02
Flavin-containing monooxygenase (*GLYMA_20G080000*) *	1.01	1.16	1.37	−1.21	−1.02	−1.01
Auxin transport genes						
NPH3 domain-containing protein (*GLYMA_14G102500*) *	1	−1.6	−2.02	−1.31	2.3	1.33
NPH3 domain-containing protein (*GLYMA*_*17G223000*) *	−2.23	−1.25	−1.06	1.16	3.56	2.22
Protein PIN-LIKES 3 (*GLYMA_03G113600*) *	−1.36	−8.51	−12.89	−1.21	3.15	2.38
Auxin efflux carrier component (*GLYMA_03G126000*) *	1.13	−2.07	−1.77	1.3	1.77	1.02
Auxin signaling genes						
Auxin-induced protein (*GLYMA_03G167400*) *	1.1	1.25	−1.01	1.02	2.07	2.86
Auxin response factor (*GLYMA_12G171000*) *	−1.09	−2.99	−2.86	1.33	1.88	−2.17
Auxin response factor (*GLYMA_12G174100*) *	3.59	−2.27	1.03	1.84	−1.58	−1.21
Jasmonate biosynthesis genes						
Jasmonic acid-amido synthetase JAR1 (*GLYMA_07G057900*) *	−1.46	−1.57	−1.64	1.19	−1.37	−1.21
Jasmonic acid-amido synthetase JAR1 (*GLYMA_16G026900*) *	4.63	−1.23	−1.37	1.25	−7.85	−4

* GLYMA_10G128700, Panther name: INDOLE-3-PYRUVATE MONOOXYGENASE YUCCA3-RELATED, Uniprot protein: I1LAM2; GLYMA_20G080000, Panther name: INDOLE-3-PYRUVATE MONOOXYGENASE YUCCA3-RELATED, Uniprot protein: I1NEK1; GLYMA_07G057900, Panther name: JASMONIC ACID-AMIDO SYNTHETASE JAR1, Uniprot protein: A0A0R4J3L2; GLYMA_03G167400, Panther name: AUXIN-RESPONSIVE PROTEIN IAA10-RELATED, Uniprot protein: A0A0R0KKH0; GLYMA_03G126000, Panther name: AUXIN EFFLUX CARRIER COMPONENT 1, Uniprot protein: K7KEN1; GLYMA_12G171000, Panther name: AUXIN RESPONSIVE FACTOR 4, Uniprot protein: A0A0R0H664; GLYMA_12G174100, Panther name: AUXIN RESPONSE FACTOR 10-RELATED, Uniprot protein: I1LTK0; GLYMA_16G026900, Panther name: JASMONIC ACID-AMIDO SYNTHETASE JAR1, Uniprot protein: A0A0R0FKJ1; GLYMA_03G113600, Uniprot protein: I1JMS6; GLYMA_17G223000, Panther name: BTB/POZ DOMAIN-CONTAINING PROTEIN NPY1, Uniprot protein: K7MND2; GLYMA_14G102500, Panther name: BTB/POZ DOMAIN-CONTAINING PROTEIN NPY1, Uniprot protein: K7M604. Genevestigator database experiment microarray IDs—GM-00305, GM-00128. ** seedlings treated with propiconazole 5 µM for 10 days. *** roots treated with propiconazole 0.1 µM for 2.5 days. **** roots treated with propiconazole 0.01 nM for 12 h. ***** roots treated with propiconazole 0.1 µM for 2 d + ECS 0.01 nM for 12 h against roots treated with propiconazole 0.1 µM for 2 d. ****** seedlings treated with propiconazole 5 µM for 10 d + BL 1 µM for 1 h against seedlings treated with propiconazole 5 µM. ******* seedlings treated with propiconazole 5 µM for 10 d + BL 1 µM for 8 h against seedlings treated with propiconazole 5 µM.

## Data Availability

Data are available on the request from the corresponding author.
